# Recovery of Phosphorus and Metals from the Ash of Sewage Sludge, Municipal Solid Waste, or Wood Biomass: A Review and Proposals for Further Use

**DOI:** 10.3390/ma16216948

**Published:** 2023-10-29

**Authors:** Sara Tominc, Vilma Ducman, Wolfgang Wisniewski, Tero Luukkonen, Gunvor M. Kirkelund, Lisbeth M. Ottosen

**Affiliations:** 1 Laboratory for Cements, Mortars and Ceramics, The Department of Materials, Slovenian National Building and Civil Engineering Institute (ZAG), Dimičeva ulica 12, 1000 Ljubljana, Slovenia; vilma.ducman@zag.si (V.D.); wolfgang.wisniewski@zag.si (W.W.); 2 Faculty of Technology, Fibre and Particle Engineering, University of Oulu, P.O. Box 8000, 90570 Oulu, Finland; tero.luukkonen@oulu.fi; 3 Department of Environmental and Resource Engineering, Technical University of Denmark (DTU), Brovej, 2800 Lyngby, Denmark; gunki@dtu.dk (G.M.K.); limo@dtu.dk (L.M.O.)

**Keywords:** critical raw materials, extraction, sewage sludge ash, municipal solid waste incineration ash, wood biomass ash

## Abstract

This review provides an overview of methods to extract valuable resources from the ash fractions of sewage sludge, municipal solid waste, and wood biomass combustion. The resources addressed here include critical raw materials, such as phosphorus, base and precious metals, and rare earth elements for which it is increasingly important to tap into secondary sources in addition to the mining of primary raw materials. The extraction technologies prioritized in this review are based on recycled acids or excess renewable energy to achieve an optimum environmental profile for the extracted resources and provide benefits in the form of local industrial symbioses. The extraction methods cover all scarce and valuable chemical elements contained in the ashes above certain concentration limits. Another important part of this review is defining potential applications for the mineral residues remaining after extraction. Therefore, the aim of this review is to combine the knowledge of resource extraction technology from ashes with possible applications of mineral residues in construction and related sectors to fully close material cycle loops.

## 1. Introduction

The European Commission (EC) has recognized the necessity to recover critical raw materials (CRMs), not only from primary but also from secondary sources as much as possible. In 2015, the document “Closing the loop—An EU action plan for the Circular Economy” expressed the EC’s aim to support the Raw Materials Information System, which would provide data on secondary raw materials (SRMs). SRMs can be sources used instead of primary raw materials. SRMs can also be a source for CRMs, which are of high economic importance due to their expected scarcity in the EU market or a high risk of supply disruption. The EU thus wishes to promote recycling these materials, especially the recovery of CRMs, as part of the move towards the circular economy [1]. This review focuses on various incineration ashes as SRMs and recovering CRMs and other resources from them.

**Figure 1:**
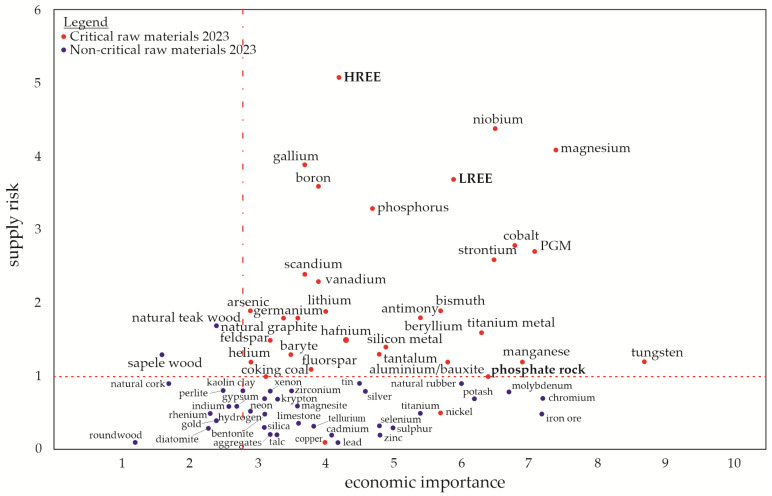
Raw materials (CRMs list from 2023) in relation to their economic importance and supply risk, redrawn based on an EU report [6]. The “critical area” is defined by a supply risk ≥ 1 and an economic importance ≥ 2.8.

One CRM is phosphorus (P), which is an irreplaceable resource and an essential nutrient for the growth of organisms [2,3]. Most P is currently extracted from phosphate rocks, which are the primary and non-renewable sources occurring in a limited number of deposits worldwide [4]. Most of the world’s phosphate rock deposits are located in Morocco, while the rest are found in China, the United States, South Africa, and Jordan [5]. These global P resources will be depleted within a few decades, which is why the EC has added P and phosphate rock to the list of CRMs shown in Figure 1 [6]. Solutions for a P recovery from various secondary resources such as ashes are hence required. Optimized use of P in agriculture and soil stabilization to prevent erosion is additionally encouraged. Rare earth elements (REEs) are another limited resource, and the EC placed them in the CRMs list due to the increasing demand for them in current industrial production (e.g., permanent magnets required for electric motors or wind energy). They are sorted into the groups of light and heavy REEs (LREEs and HREEs, respectively) [6,7].

Even apart from CRMs, there is a fundamental dependence on metals in different industries. Non-ferrous base metals, such as Cu, Zn, and Pb, are some of the most used metals worldwide, only exceeded by Fe and Al. All these elements are necessary for our current society and infrastructure. Projections predict an increase in the demand for Cu by 140%, Zn by 81%, and Pb by 46% compared to the 2010 demand until 2050 [8]. By the end of the 21st century, the projected demand is even higher for Cu and Zn (330% for Cu and 130% for Zn) [8]. At the same time, the expected depletion years for these elements (the year when the cumulative primary production exceeds the reserves) can be reached before 2025–2038 without the peak year of primary production being reached [8]. Consequently, used ore grades become lower, which typically leads to an increased energy demand for extraction, e.g., for fossil fuels, resulting in increasing greenhouse gas emissions for constant production [9,10]. This calls for developing new economically viable mining methods and extraction strategies applicable to SRMs. The use of elements in products usually dilutes their concentration [11], whereas incinerating end-of-life products causes the accumulation of elements in incineration residues, which can hence be an important source for improving the circularity of elements otherwise deposited in landfills.

Table 1 compares the concentrations of P and selected heavy metals reported in various ashes with typical concentrations in mined ores. Sewage sludge ash (SSA) is a rich source of P, while municipal solid waste incineration fly or bottom ashes (MSWI-FA, MWSI-BA) are rich in metals, especially Zn, Pb, and Cu. Wood biomass fly or bottom ashes (WB-FA, WB-BA), on the other hand, do not have a very high P content but contain essential micronutrients for plants as well as Na [12]. The composition of the biomass varies depending on the combusted biomass type [12,13,14,15]. REEs are generally not enriched in these ashes in comparison to the concentrations found in the Earth’s crust [7]; combined REE concentrations of 88–124 mg/kg were found in MSWI-BA and averaged 54 mg/kg in MSWI-FA sampled in Italy [16]. Biomass ashes contain even lower combined REE concentrations of, e.g., ~27 mg/kg [17]. Recovering REEs from such ashes may still become economical as they can be co-extracted with the other resources in Table 1, and their demand is expected to reach a ~4.4% annual growth rate globally by 2026 [18] and rise by 2600% over the next 25 years [19]. Despite extensive, mostly lab-scale research efforts on REE recycling, less than 1% were recycled in 2011 due to inefficient collection, technological problems, and a lack of incentives [20]. Achieving a circular economy and a truly sustainable society must include the recovery of REEs from SRMs with low concentrations [7].

Legislation can be a powerful tool to promote recycling. Following the legislative developments in Switzerland and Germany, Austria has also adopted legislation for a mandatory P recovery from SSA. In 2022, the Swiss Federal Council published an Ordinance on the Avoidance and the Disposal of Waste (SR 814.600), where P must be recovered and recycled from SSA (Section 3, Art. 15, in force since 1st Jan. of 2019) and metals recovered from MSWI filter ash (Section 3, Art. 32) [32]. There are also requirements and limit values for using waste as a raw material in this Ordinance (Annex 4, in force since 1 January 2022) [32]. Denmark has the Statutory Order No. 1672/2016 on the use of residual products, where MSWI-BA is on the list to replace primary raw materials [26]. However, the ash must meet quantitative criteria regarding the content and leachability of certain inorganic substances [26]. The Statutory Order No. 732/2019 on the application of biomass ash in agriculture additionally determines the extent to which biomass ash can be requested as a replacement for commonly used fertilizers or soil improvers (i.e., which types of biomass ash are allowed, the limit values for the content of heavy metals, the maximum amount of allowed ash and its reactivity) [26]. The Finnish legislation on fertilizers has also been modernized, as a new Fertilizer Act 711/2022 came into force in July 2022 [33].

## 2. Recovery of Resources from Selected Ashes

The extraction of specific components from ashes is a wide field as the ash properties vary depending on the fuel type and composition, ash fraction, and power plant processes, and various components are of interest for extraction. The ashes selected for this review are rich in siliceous phases, macronutrients such as P, K, Ca, Mg, or S necessary for plant growth [34], and contain trace amounts of Fe, Zn, and/or Cu. The content of potentially toxic elements is also very important, but some of these are also necessary micronutrients for plant growth. The main elements of interest in this review are P and the potentially toxic elements Zn, Pb, Cd, Cr, and Cu. They are usually extracted using various organic/inorganic acids, chelating agents, or basic solvents, as well as electrochemical methods such as electrodialytic separation (EDS).

### 2.1. Overview of Extraction Technologies

Various techniques, such as wet extraction, thermochemical, and electrochemical methods, have been developed to extract or recover metals from different ashes [35,36]. This overview also covers processes at the technology readiness level (TRL) 7 (see Section 2.2) using wet extraction methods. Wet chemical extraction is the most widely used method for extracting P from various ashes due to its high recovery rate, low cost, and procedure simplicity. Choosing the right extractant is very important. Common examples are inorganic acids such as sulfuric acid (H_2_SO_4_) [2,4,22,23,24,25,37,38,39], nitric acid (HNO_3_) [4,23,25,37,39], and hydrochloric acid (HCl) [4,15,40,41]; organic acids and chelating agents such as citric acid (CA) [25,37,38,39], oxalic acid (OA) [4,22,25,37,38,39], lactic acid (LA) [37], ethylenediaminetetraacetic acid (EDTA) [25,38,39], and ethylenediamine tetra (methylene phosphonic acid) (EDTMP) [25,38,39]; and bases such as NaOH [4]. Novel chelating agents for ashes could also be bisphosphonates [42].

The most commonly used inorganic acid and the cheapest extractant on today’s market is H_2_SO_4_ [23]. Its main advantages are easy transportation due to its low volatility, the possibility of concentrating up to 98%, and ensuring less co-dissolution of heavy metals, especially Pb [2,4]. Other inorganic acids, such as HNO_3_ [4,23,25,37,39], HCl [4,15,40,41], and H_3_PO_4_ [5], have also been used. HCl may facilitate the occurrence of unwanted complexation reactions [2], and H_3_PO_4_ is comparably expensive [5]. Organic acids are usually chosen in research for their reduction properties (especially OA, which is the strongest naturally occurring organic acid) and for their environmentally friendly production (CA and LA) [22,25,37,38,39]. OA is the most efficient P extractant among the organic acids as it combines a high P extraction efficiency with a relatively low co-extraction of heavy metals; however, H_2_SO_4_ has an economic advantage over OA due to the lower costs for optimal P extraction [4,25].

Chelating agents have a marginal effect on the morphology and particle size distribution of ashes and are less effective for P recovery compared to inorganic and organic acid extractants due to their high affinity to metal ions, resulting in partial dissolution of P [34,39]. EDTA performed better than EDTMP for trace elements such as Zn, Pb, or Cd, so EDTA could be used ahead of the P extraction to remove significant quantities of metals without leaching P [38]. Fang et al. also published a study where a combination of EDTA and H_2_SO_4_ was used but was ineffective for P extraction [43]. The use of Cyanex, a highly stable P-based chelating extractant, is effective for the extraction of heavy metals (Zn, Pb, and Cd) from leachates after wet extraction [27,44]. However, the selective separation of REEs requires very specific kinds of chelating agents, where bisphosphonates could be a promising option [42]. They contain a carbon center with two phosphonate groups (PO(OH)_2_) and two other substituents (e.g., a hydroxyl group and a carbon chain with a primary amine group) [45]. Bisphosphonates have been immobilized on nanoporous silicon and then applied to selectively recover Sc from a highly complex ore sample leaching solution [42]; a similar approach could be applied to ashes. The desorption of Sc from bisphosphonates can be conducted by using acids such as H_3_PO_4_ and H_2_SO_4_ [42]. Bisphosphonates can also be used for a relatively selective recovery of other REEs, but information remains scarce [46].

Alkali-metal bases such as NaOH dissolve almost no heavy metals, mainly due to the high pH of around 13 at the end of the extraction procedure. They are also ineffective for P extraction because Ca-phosphates show poor solubility in alkaline environments (especially when the molar P/Ca ratio is lower than one), while Al- and Fe-phosphates are highly soluble in such media [4,24].

Optimal process conditions require combining the highest P extraction efficiency, the lowest possible co-extraction of heavy metals, and the lowest possible operating costs. Additionally, variables such as the extractant type and its concentration, the contact time (optimally 2 h [2,22,24,38,39]), the liquid/solid ratio (optimally 20:1 [2,23,24,38]), the extraction temperature and ash composition significantly affect the P extraction efficiency [2,22,24]. Longer extraction times (e.g., one week compared to 2 h) result in a lower P extraction efficiency and more heavy metal leaching [23]. It is also necessary to consider the variability of certain elemental contents, e.g., the P content in SSA can be partially attributed to differences in wastewater treatment systems and incineration conditions [47]. The sampling period also appears to influence its content, as, e.g., a lower P content has been measured in the summer months while higher contents were measured in February and March [48]. This can be explained by the different food habits and leisure activities of people in different seasons [48]. The recovery of P by wet chemical extraction can be effectively applied to different types of SSA, as they contain higher amounts of P [2,4,22,23,24,25,37,38,39]. Ashes from wood biomass and MSWI are not as rich in P as SSA, but they are rich in Zn, which is also important to recover [40,44,49,50,51].

An overview of the extractants used to extract P, Zn, Pb, Cu, Cr, and/or Cd from the selected ashes is presented in Table 2, along with the respective achieved extraction rates. In the case of P, the highest extraction rate of 100% was achieved using H_2_SO_4_ [23], HNO_3_ [23,37], and OA [22], while the most effective extractant for Zn was HCl, reaching 86% [41]. The highest reported extraction rate in the analyzed literature for Cr was 58% [39], 74% for Cu [38], 62% for Pb [40], and 97% for Cd [40]. Although chemical extraction achieves high efficiencies, it requires further purification and the treatment of insoluble acid residues [22,34,38,52,53]. It also often requires undesirably large amounts of acids, encouraging researchers to develop alternative methods [35]. One alternative for achieving high extraction rates of Zn (around 90%), Pb, and Cd from ash is the thermochemical method, but concerns regarding its operating costs, high energy input, and equipment lifetimes have been voiced [52,53,54]. KCl, MgCl_2_, and CaCl_2_ are often added in the thermochemical process, where high concentrations of chlorine compounds can be extremely corrosive [53,55,56]. Increasing the treatment temperature led to higher Pb and Zn removal rates [55], and above 1400 °C, the thermal removal of heavy metals also enabled the separation of Fe, increasing the bioavailability of P in the ash [53]. The most promising one-step extraction method is EDS, whose main advantage over other techniques is the ability to separate P from the remaining waste and remove heavy metals from ash in one process [3,57,58,59,60]. Here, electromigration separates the component by transporting P towards the anode and heavy metals mainly towards the cathode, a very important aspect considering mixed component wastes [57]. After the treatment, P is recovered from the anolyte by filtration to separate the liquid from the remaining solids, and the heavy metals are solubilized in the catholyte [59]. However, the EDS process is time consuming [57,58,60,61], and the operating costs are relatively high [52,57,62].

### 2.2. Methods Applied on an Industrial Scale

Prototype plants are already implemented or under construction (i.e., at TRL7) for the wet extraction of P from SSA (EasyMining, Uppsala, Sweden), salts and Zn from MSWI ashes (Stena Recycling, Gothenburg, Denmark), Zn from MSWI ashes (RENOVA, Göteborg, Sweden) and the full-scale commercially available process FLUWA/FLUREC operating in Switzerland for the recovery of metals. Additional plants recovering P from SSA using H_3_PO_4_ are operating in Germany, Switzerland, and Japan. These wet extraction processes at the high TRL level are based on using waste acid from nearby industries or from the wet scrubber of the incineration plant itself. A wet extraction method using HCl and lime for recovering commercial P, Fe, and Al products called Ash2Phos was developed by EasyMining, Sweden. The process has recovery rates of 90–95% for P, 60–80% for Al, and 10–20% for Fe from SSA [63]. Simultaneously, the heavy metal content in connection to P is reduced by at least 96%, making it a very pure and clean fertilizer product [63]. SSA is dissolved in HCl at 40 °C, and the P, Fe, and Al are separated as pure Ca_3_(PO_4_)_2_, FeCl_3_, and NaAlO_2_ [63]. The separation process is based on chemical precipitation steps in a unique combination, and the solution is later neutralized to remove heavy metals. CaO is used during the precipitation steps and for neutralization. The produced phosphorus–calcium-rich product (Ca_5_(PO_4_)_3_OH) contains a minimum of 16.5% P and 35% Ca and can be used as raw material for feeds or fertilizer applications [63]. The Fe and Al products can be reused in wastewater treatment plants [63]. After the treatment, non-dissolved SSA is filtered, washed, neutralized, and called “silicate sand” (48.3% SiO_2_, 22.9% Fe_2_O_3_, 7.2% Al_2_O_3_), which is potentially useable as a partial cement replacement in mortars after milling [64]. A full-scale plant able to annually treat 30,000 tons of SSA is under construction in Sweden, and plants in Germany are also under development [63].

The recovery of metals from MSWI-FA is achieved by the FLUWA/FLUREC processes developed in Switzerland by AIK Technik AG, as well as by the HaloSep process in Denmark developed by Stena Recycling. In 2018, >60% of the MSWI-FA in Switzerland was treated with the FLUWA process [39], which is based on wet extraction by adding acidic (HCl) and neutral (NaCl) waste scrub water to MSWI-FA where 60–80% Zn, 80–95% Cd, 50–85% Pb, 50–85% Cu can be extracted [40,65]. The metal-enriched filtrate obtained after FLUWA needs to be further processed to recover the metals, either by leading the filtrate to a wastewater treatment plant to precipitate a metal hydroxide sludge that can be recovered in smelting plants or by the FLUREC process, which allows a high-purity Zn recovery. Cd, Pb, and Cu are separated by reductive separation (cementation) using Zn powder as a reducing agent [65]. This cement, with a high Pb content of 50–70%, can be sent directly to a lead smelter where metals are recovered in the Pb production process [65]. Zn is removed from the remaining liquid by solvent extraction, followed by electrowinning to recover high-grade Zn (>99.99% Zn), which can be sold [66]. The remaining FA particles (filter cake) are currently landfilled.

The HaloSep process is another wet extraction method using a HCl scrubber liquid and MSWI-FA, which produces brine and a neutralized and washed FA. The resulting residues from the process are a stabilized FA, a metal fraction, and a brine solution. The metals are precipitated from the brine into a filter cake containing up to 38–40% Zn, which can be recovered at smelters. The remaining brine contains salt products (CaCl_2_, NaCl, KCl) useful for industrial applications. The treated FA complies with the European leaching limits for acceptance in landfills [65] but can also be used in construction [67]. A full-scale HaloSep plant is operating at the incineration plant Vestforbrænding in Copenhagen, Denmark, and plants in other countries are under exploration [67].

The RENOVA process also uses a HCl scrubber liquid for Zn leaching but differs from HaloSep by using an acidic pH in the process and re-incineration of the leached ash to destroy dioxins. Up to 70% Zn was leached in pilot scale studies, and NaOH-precipitated filter cake contained 80% Zn(OH)_2_ [68]. Re-incineration studies showed that more than 90% of the leached ash was converted into bottom ash [68]. There are plans to build a demonstration plant in Sweden [69].

Additional plants recovering P from SSA using H_3_PO_4_ are, e.g., a sewage sludge incineration plant in Werdohl, Germany, which uses the Remondis TetraPhas process [52]. It consists of leaching P from SSA by H_3_PO_4_ and purifying the P-concentrated acid leachate, allowing an 80% P extraction. The product, called RePacid, mainly contains H_3_PO_4_ and can be directly used by the industry [52]. Another solvent-extraction process called Phos4life was designed in the canton of Zürich, Switzerland, where the main product is technical H_3_PO_4_ (74%). Here, P is extracted from SSA by H_2_SO_4_, and more than 95% of it can be recovered from SSA in the form of H_3_PO_4_ [52]. Another well-known P production company is Nippon Phosphoric Acid Co., Ltd. (NPA) in Achi, Japan, where the H_3_PO_4_ is also obtained through a wet extraction process followed by filtration and purification. Gypsum (CaSO_4_·2H_2_O), with possible applications in cement, plasterboard, or soil improvement, is a by-product of this process [52].

### 2.3. Economic Assessment of Alternative Extraction Methods

In addition to achieving an optimal environmental profile for the extracted resources, the economic costs of recovering secondary raw materials are also of great importance, as both environmental and economic costs influence their acceptance in society [35]. Although high percentages of P extraction can be achieved by acid wet extraction of P, the acid demand is very high [70]. An example of acid demand is given by Donatello et al., where an optimized procedure showed that 368 kg of 98% H_2_SO_4_ was required for 80–100% P extraction from 1 ton of SSA [71]. Handling and transporting such large quantities of acid is not easy in all urban environments [70]. Moreover, the simultaneous extraction of heavy metals and P [71] requires a second separation step to support the European Green Deal, which promotes the recycling of limited resources while emphasizing the goal of zero pollution [70]. For this purpose, the development of alternative technologies, such as thermochemical methods, is supported. Products recovered by thermochemical methods are still ash, and the separated heavy metals represent only a small part of the ash, which is suitable for transport [72]. However, the energy consumption and high capital costs of the alternative methods still need to be optimized compared to the traditional wet chemical method [3,34,36]. In 2016, Egle et al. reported that the cost of recovering P from SSA using the wet chemical method is about 5–6 EUR/kg of P, whereas using the thermochemical method, the cost is about 2–3 EUR/kg of P [73]. However, the thermochemical method is closely related to the energy price, and with the current increasing energy prices, the cost of the thermochemical method has certainly increased [72]. Therefore, alternative extraction methods, such as microwave-assisted acid extraction, can reduce the total cost by up to 76% for MSWI-FA and up to 52% for MSWI-FA compared to traditional metal extraction by heating [35]. This cost analysis considered the cost of acid/chemicals, energy consumption, miscellaneous costs, and other laboratory costs for processing a given amount of ash [35]. In addition, alternative technologies, such as electrochemical methods, are still in the development and optimization phase, so there are not many analyses of their economic performance.

## 3. Potential Uses of Solid Residues of Ashes

Waste incineration is steadily increasing in Europe, but there are environmental concerns about the solid residues that require pre-treatment and are usually landfilled [74]. There are possibilities to use as-received or pre-treated ashes in agriculture, soil stabilization, and the building sector as the following:

- Supplementary cementitious materials (SCMs);
- Precursors for alkali-activated materials (AAMs);
- Artificial fillers or fine aggregates;
- Additives in clay-based materials;
- Precursors for carbonated products.

The use of waste ash in construction materials has attracted many researchers in recent years [74]. However, a good understanding of their chemical, physical, and microstructural characteristics is necessary for their full-scale use [75]. The question of how to keep the ash characteristics constant when heterogeneous materials such as sewage sludge, MSW, or varying types of biomasses are incinerated is especially important for their large-scale utilization.

AAMs/geopolymers are synthetic materials obtained by alkaline activation of Si- and Al-rich materials and specific industrial wastes [76]. AAMs are alternative cementitious or ceramic-like binders used as alternative construction materials and for the solidification/stabilization of various waste streams [77]. As cement production is among those human activities most generating CO_2_ emissions, sustainable development of the building industry requires three approaches: using renewable energy, using recycled products, and replacing cement [78]. Every 600 kg of cement causes about 400 kg of CO_2_ to be released into the atmosphere [79,80,81,82,83]. Therefore, the potential applications of different ashes as SCMs have been studied for decades [84] but recently with a focus on bio-based ashes [80].

Another very promising research topic in the cement and concrete industry is carbonation utilizing CO_2_ sequestration: a low-tech approach to the carbon capture and storage process that mainly involves the reaction of CO_2_ with Ca-containing materials to form Ca carbonates [85,86,87]. Potential sources from waste streams are ashes containing a certain amount of Ca and Mg compounds, especially WBA [88,89,90].

### 3.1. Potential Use of SSA

Sewage sludge is the most common and continuously generated by-product of wastewater treatment, containing the 2nd highest amount of P after bone meal [3,5,47]. It has a great potential for P recovery after an appropriate thermal treatment [2,4,22,23,24,25,37,52,53]. Sewage sludge has been directly used as agricultural fertilizer for decades, but its limitations are increasing all over the world due to the high contents of heavy metals, organic pollutants, and micro/nanoplastics. Hence, its incineration is considered to be the best way for disposal [53]. The incineration of sewage sludge at about 850 °C is widely used in the EU and is currently the most efficient method, reducing the volume by 90% (the mass by 70%) and removing organic pollutants and pathogens [47,52,59]. The resulting SSA contains 4–12 wt% of P, usually in the form of AlPO_4_ and Ca_3_(PO_4_)_2_, which are poorly bioavailable [26]. SSA also contains Fe and potentially toxic trace elements such as Zn, Pb, Ni, Cr, and Cd and is mostly landfilled [5,23,48]. Pre-treatment is required to prevent the loss of this potential P source and aims to increase the bioavailability of P and remove heavy metals, which often exceed the legal limits for fertilizer production, see Table 3 [24,48,74,91]. With, for example, innovative EDS, 80–90% of the P can be recovered while also achieving a low content of heavy metals [58,59,60]. A high concentration of CaO and SiO_2_ in the SSA after P extraction is the main reason for using SSA as a building material component [5].

SSA is a material comparable to lightweight sand and is less dense than Portland cement [47]. It consists of porous particles with irregular shapes, which is not ideal for its classification as a potential cementitious material [47]. It should be noted that the extraction of P with H_2_SO_4_ produces CaSO_4_, which negatively affects the cement properties [4]. Using OA as an extractant produces Ca oxalate, which does not have this negative effect [4]. SSA typically contains an elevated amount of about 14% Al_2_O_3_ compared to the ca. 5% in Portland cement, indicating a natural suitability for use in aerated concrete [47]. The high Al_2_O_3_ content in SSA may also benefit the chloride attack resistance in concrete applications due to the chloride binding capacity of amorphous Al_2_O_3_ [47]_._ SSA can be used as a possible cement replacement material, but it requires a pre-treatment due to the undesirable effects of the contained heavy metals and P recovery. It has the potential to replace cement in mortars [23,92] or partially replace clay in bricks [59]. Due to the small grain size, SSA is also suitable as a filler or fine aggregate component in mortar and concrete, where the effects on strength performance have been shown to be manageable for SSA contents up to 15 wt% [47,49,93]. Research on the reuse of SSA as an aluminosilicate precursor material for alkali activation/geopolymerization has also recently begun [77,94,95,96].

### 3.2. Potential Use of MSWI Ashes

**Figure 2:**
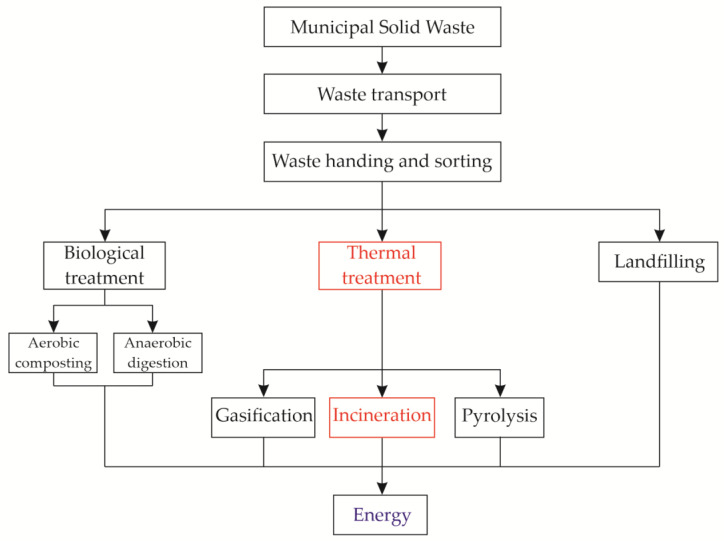
Different treatments for MSW management; redrawn based on [97].

MSW contains wood but also paper, plastic, glass, and textile scrap material, which cannot be degraded naturally. In the last few decades, the total mass of MSW has increased drastically due to rapid urbanization and an increased world population. This has encouraged many countries to properly dispose of this waste [74,97]. The so-called “green economy” has begun, encouraging waste reduction, reuse of materials through recycling or recovery, and supporting sustainability [97,98]. Several different treatments of MSW have been developed, as shown in Figure 2. The gases produced by the natural decomposition of MSW in landfills represent 18% of the energy production from biogas in the EU [97,99]. Nowadays, one effective and popular method is the incineration of MSW due to the volume reduction in MSW by 90% recovery of heat/energy. Two main residues are produced by incineration: around 80 wt% MSWI-BA and around 20 wt% MSWI-FA [27,51,97,100,101,102].

MSWI-BA is classified as non-hazardous waste and mainly consists of amorphous SiO_2_, Al_2_O_3_, and CaO; its exact composition varies from incineration plant to incineration plant and even from batch to batch within a single incineration plant [97,100]. MSWI-BA is commonly utilized in road construction [103] and can be an alternative lightweight aggregate [97] or an alternative material for cement production [104,105]. One of the advantages of using MSWI ash as cement raw material is the reduction in CO_2_ emissions [104]. MSWI-FA is classified as a hazardous waste as it contains soluble salts, dioxins, and a significant amount of heavy metals such as Zn, Pb, and Cd [40]. Due to the presence of potentially leachable contaminants harmful to the environment and human health, landfill sites are becoming fewer, and the possibility of utilizing MSWI-FA has attracted many researchers [27,35,50,51,61,101,102,106]. Sekito et al. reported a 2-fold higher content of Zn and Pb in MSWI-FA compared to MSWI-BA, while the content of Cd was even 13-fold higher [107]. Therefore, MSWI-FA must be pre-treated before further use, and much research has focused on how to extract and recover various metals from it. In MSWI-FA, the pH has a significant effect on the removal efficiency of heavy metals [50]. Many metals have a high solubility at low pH levels, so using strong acids as the extractant is necessary. As MSWI-FA is alkaline, alternative methods are desired to avoid the consumption of large acid volumes. A new microwave-assisted acid extraction method has recently been developed [35]. Significant advantages of this method are lower costs, shorter processing times, and better efficiency of metal extraction compared to conventional heating [35,108,109,110].

Electrodialytic (ED) treatment is another innovative method also used for contaminated SSA [59]. It reduces the content of heavy metals and salts and increases the reactivity of Si and the Si/Al ratio [61,111]. Such a pre-treatment method can make MSWI-FA into a potential precursor in geopolymers based on AAMs that can naturally trap heavy metals inside its matrix. As MSWI-FA is alkaline (having a pH of around 11), ED treatment results in an acidic pH, similar to the common natural precursor in geopolymers, i.e., metakaolin. The combination of MSWI-FA pre-treatment and initiating up to 20 wt% of MSWI-FA in geopolymers achieves the lowest metal leaching and a high compressive strength, making it a potential construction material [61,111]. Thus, the use of geopolymerization for hazardous waste not only contributes to the best technological practices and legal provisions but is also ecologically efficient [112]. Studies have shown that the use of raw MSWI-FA is also possible but achieves lower compressive strengths than that of AAMs prepared with slag or pulverized FA [101]. The reason for the low compressive strength could be its variable mineral composition because MSWI-FA contains less SiO_2_ and Al_2_O_3_ than slag or pulverized FA and has a large specific surface area due to a high proportion of small particles [101].

MSWI-FA has also been studied as a potential replacement for cementitious materials, but adding it to cement-based products means that technical and environmental requirements such as sufficient strengths, disabilities, and leaching limits of heavy metals must be met [113]. The main problem with using MSWI-FA as a cement substitute is the presence of leachable toxic heavy metals and a high salt content. It is beneficial to use water washing and mechanochemical [113] or ED pre-treatments [114] to improve its performance before it is used in mortar, concrete, or bricks. The mechanochemical processes can stabilize the heavy metals and activate the MSWI-FA, allowing it to partially replace Portland cement in building materials [113], while an ED treatment can remove heavy metals and soluble salts from the MSWI-FA suspension which is thus decontaminated [96,115]. As an ultrafine material, MSWI-FA is also a potential substitute for clay in bricks, which should stabilize the heavy metals, reduce raw material imports, and, at the same time, conserve primary clay resources [115]. Studies have shown that fired bricks with an addition of 2.5–5 wt% treated MSWI-FA may be feasible [96,116,117].

### 3.3. Potential Use of WBA

WBA results from wood biomass combustion generating WB-BA, collected from the bottom of a combustion chamber, and WB-FA, which is subdivided into fine fly ash (particle size <1 µm, collected from electrostatic or bag house filters) and coarse fly ash (particle size >1 µm, collected from the cyclone or boilers) [83,118,119]. CaO and SiO_2_ are generally the major chemical components in WBA, while other compounds such as Al_2_O_3_, Fe_2_O_3_, K_2_O, Na_2_O, MgO, P_2_O_5_, or SO_3_ occur in lower amounts [15,75,120]. Minor contents of As, Cd, Cr, Cu, Pb, and Ni have also been detected [15,75]. Significant differences in the content of volatile heavy metals occur amongst the ash types and are the main concern when using WBA. A higher concentration of heavy metals was measured in WB-FA compared to WB-BA, as heavy metals are more concentrated in smaller particle size fractions (<75 µm) [12,14,118,119]. The particles of the fine fly ash fraction are also lighter and smaller, making them easy to inhale and a health risk, e.g., Cd accumulates in the kidneys and affects bone density [121].

The chemical composition of WB-FA also differs from coal FA, as WB-FA usually contains more alkali elements and less Al [118]. Nutrients, such as P and Mg, are primarily found in the WB-BA and coarse WB-FA. WB-FA shows significantly lower Cd concentrations compared to MSWI-FA [12,15,28,29]. The chemical and physical properties of WBA depend on the combustion technology, the heat treatment temperature, the tree species, and the geographic location; however, other factors, such as soil conditions, climate characteristics, and storage methods, also influence its properties [12,13,14,15,82,83,118,121,122].

Since wood biomass is considered to be a CO_2_-neutral renewable energy source, it is environmentally desirable to use WBA in the construction industry [119,123]. This would not only reduce rising disposal costs, 70% of WBA still ends up in landfills, but also preserve natural resources and reduce greenhouse gas emissions [119,124]. WBA has the potential to be used in various construction areas: as a partial replacement of aggregates or mineral admixtures in concrete [118,124], as a partial replacement of raw materials for clinker [118,119], as a filler/partial sand replacement material in cement-based materials [13,28], in brick production [13], road construction [13] and others. Proper storage and transport conditions are important for WBA use in cementitious composites, as carbonation and hydration can occur suddenly during these procedures in wet circumstances and thus strongly determine the quantity of CaO and other carbonate elements [13]. It is also very important to find the optimal cement/ash ratio so that the strength of the cement composites remains sufficient [13,125]. Replacing up to 45% of cement with WB-FA has been described as suitable for construction purposes; however, WB-FA has more potential as a filler material than as a cement replacement material in construction [13,82]. Most studies report that the optimal content of WB-FA and WB-BA to replace part of the cement in mortars is 10 wt% [13,79,80,126]. It has been reported that adding WBA generally lowered the mechanical properties of bricks [127,128], but many clay mixtures still fulfilled the required strength parameters. Another emerging use of WBA is as a feedstock material for CO_2_ utilization via mineral carbonation [129,130]. Even though the CO_2_ uptake capacity is limited, it can be improved as, e.g., mechanochemical activation has doubled its capacity [131]. Biomass ash is also a low-cost medium that does not need to be transported to a plant and can be used for a low-tech sequestration approach [86].

The suitability of coal combustion FA for the production of AAMs has been proven many times due to its suitable chemical composition and the large volume available [132,133]. It is influenced by the coal properties, the combustion technique, and the waste handling. WB-FA can similarly be used as a complete or partial replacement material when preparing geopolymer mortars, which could reduce the cost of geopolymer source materials and avoid the cost of WBA landfilling [76,132]. Replacing coal combustion FA by up to 20% WBA in the binder mass resulted in better strength and porosity properties than the control mixture after 3 and 7 days of aging. After 28 days, the geopolymer containing 10% WBA was the only one to show a higher compressive strength and a lower total porosity than the control mixture [132].

Using WBA as a forest fertilizer also has potential; however, future research should focus on the effect of trace element solubility on the natural leaching processes in forest soil [12,134]. Another important factor preventing the use of WBA on certain soil typologies is its alkaline pH (usually higher than 12) [135]. Accordingly, Pasquali et al. proposed a technology to stabilize heavy metals in WBA and lower their pH based on the use of other by-products (coal FA, rice husk ash, and MSWI-FA) [135]. MSWI-FA has a similar pH as WBA and is a source of leachable heavy metals, while its P concentration is low. Ca-rich coal FA was used in the stabilization procedure, while rice husk ash was chosen as a heavy metal stabilizer due to its amorphous silica content. Wolffers et al. recently reported the recovery of heavy metals from WB-FA based on acid leaching, a process also applied to MSWI-FA [15]. In Switzerland, the disposal of WB-FA in landfills will be prohibited in 2023 due to the elevated concentrations of very toxic Cr(VI) and other heavy metals [15]. The FLUWA process represents a promising method for managing WB-FA [15].

## 4. Conclusions

This literature review focused on the technologies available to recover valuable elements, such as P and the selected heavy metals Zn, Pb, Cd, Cr, and Cu, from MSWI, SSA, and WBA. The wet chemical extraction method is used for P extraction, with H_2_SO_4_ as the most common extractant. Other methods are needed to extract resources from ashes in locations where waste acid is not easily available. Electrochemical technologies are beneficial alternatives because their major input is an electrical direct current, which can be directly gained from renewable sources, and the extraction can be performed in periods with excess grid energy. Nevertheless, future studies should deal with the duration of the electrodialytic process and reduce energy consumption.

An overview of the potential use of incineration ash as secondary raw material was provided, confirming their potential in various fields, with or without a pre-treatment. Pre-treated SSA can be used as a substitute material for cement, as a filler or fine aggregate component in mortar and concrete, can partially replace clay in bricks, or can be reused as a precursor material for geopolymerization. Non-hazardous MSWI-BA is commonly used in the construction industry as an alternative lightweight aggregate, as an alternative material for cement production, or in road construction. The hazardous MSWI-FA must be pre-treated before further use in geopolymers or as a substitute material for cementitious material or clay in bricks. For WBA, it is environmentally desirable to use it in the construction industry or for CO_2_ utilization through mineral carbonation.

## Figures and Tables

**Table 1:** Minimum and maximum contents of P and selected heavy metals in treated SSA, MSWI-FA, MSWI-BA, and wood biomass fly and bottom ashes (WB-FA, WB-BA) compared to typical ore concentrations.

Element	SSA [2,4,21,22,23,24,25]	MSWI-FA [15,26,27]	MSWI-BA [26]	WB-FA [12,28,29]	WB-BA [28]	Typical Ore Concentration [30,31]
P (g/kg) *	35–99	4–5	-	10–23	8–17	110–160
Zn (mg/kg)	895–2823	9000–70,000	610–7800	446–2274	74–234	50,000–150,000
Pb (mg/kg)	70–460	5300–26,000	100–13,700	11–177	5–80	300,000–400,000
Cu (mg/kg)	423–839	600–3200	190–8200	89–161	65–111	5000–20,000
Cr (mg/kg)	78–460	140–1100	23–3200	18–101	25–70	310,000
Cd (mg/kg)	4–126	50–450	0.3–70	7–16	0.1–0.5	1000–10,000

* Calculated based on the P_2_O_5_ content.

**Table 2:** Summary of the extractants used to extract P, Zn, Pb, Cu, Cr, and/or Cd from selected ashes (marked with the x) with corresponding extraction rates (in %). The analyzed literature does not contain such extraction rates for WBA.

Extractant	Conc. (mol/L)	P	Zn	Pb	Cu	Cr	Cd	SSA	MSWI	WBA	Reference
H_2_SO_4_	0.05	>95						x			[22]
	0.1	88.3						x			[24]
	0.19	100						x			[23]
	0.19	~88						x			[23]
	0.2	92						x			[2]
	0.2	94						x			[38]
	0.25	93	~36	~1	~38	~5	~28	x			[4]
	0.4	96.4						x			[37]
	0.5	>70	~42	38.4	~40	57.7	50	x			[39]
	0.5	74	~42		~40			x			[25]
HNO_3_	0.3	89	~32	~24	~36	~5	~27	x			[4]
	0.4	100						x			[37]
	0.5	>70	~36	40	~38	~52	~6	x			[39]
	0.5	~71	~36					x			[25]
	1.5	~80	16	56				x			[23]
	1.5	100	71	47				x			[23]
HCl	0.3	98.8	~32	~30	40	~5	~28	x			[4]
	0.5	>95						x			[41]
	1.0		75	1			71		x		[40]
	1.0		~58	~1			40		x		[15]
HCl + H_2_O_2_	1.0 + 9.8		68	62			97		x		[40]
Citric acid (C_6_H_8_O_7_)	0.2	~80						x			[38]
	0.4	59.3						x			[37]
	0.5	>70	~23	13.3	~16	~25	~7	x			[39]
	0.5	72	~23		~16			x			[25]
Oxalic acid (C_2_H_2_O_4_)	0.05	100						x			[22]
	0.2	>95						x			[38]
	0.55	95.4	37	~1	37	~8	~13	x			[4]
	0.4	100						x			[37]
	0.5	>70	56.9	4	65.8	~53	~13	x			[39]
	0.5	74	56.9		65.8			x			[25]
Lactic acid (C_3_H_6_O_3_)	0.4	28.4						x			[37]
EDTA (C_10_H_16_N_2_O_8_)	0.02	~20						x			[38]
	0.05	<30	~14	37	~5	~42	~6	x			[39]
	0.05	~24	~14		~5			x			[25]
	0.05	~40						x			[38]
EDTMP (C_6_H_20_N_2_O_12_P_4_)	0.05	<30	~10	~22	~9	~26	~6	x			[39]
	0.05	~13	10		~9			x			[25]
	0.05	~25						x			[38]
NaOH	0.5	40	~3	~3	~2	~2	~4	x			[4]

**Table 3:** Legal limits for trace elements in EU fertilizing products (in mg/kg), adapted from the EU regulation [86].

Element	Organic Fertilizer	Organo-Mineral Fertilizer	Inorganic Fertilizer
As	40	40	40
Cd	1.5	3	3
Cr	2	2	2
Cu	300	600	600
Hg	1	1	1
Ni	50	50	100
Pb	120	120	120
Zn	800	1500	1500

## Data Availability

Not applicable.

## References

[1] European Commission (2015). Closing the Loop—An EU Action Plan for the Circular Economy, Communication from the Commission to the European Parliament, the Council, the European Economic and Social Committee and the Committee of the Regions, COM(2015) 614 Final. https://eur-lex.europa.eu/legal-content/EN/TXT/?uri=CELEX:52015DC0614.

[2] Boniardi G., Turolla A., Fiameni L., Gelmi E., Malpei F., Bontempi E., Canziani R. (2021). Assessment of a simple and replicable procedure for selective phosphorus recovery from sewage sludge ashes by wet chemical extraction and precipitation. Chemosphere.

[3] Kwapinski W., Kolinovic I., Leahy J.J. (2021). Sewage sludge thermal treatment technologies with a focus on phosphorus recovery: A review. Waste Biomass Valorization.

[4] Luyckx L., Geerts S., Van Caneghem J. (2020). Closing the phosphorus cycle: Multi-criteria techno-economic optimization of phosphorus extraction from wastewater treatment sludge ash. Sci. Total Environ..

[5] Cieślik B., Konieczka P. (2017). A review of phosphorus recovery methods at various steps of wastewater treatment and sewage sludge management. The concept of “no solid waste generation” and analytical methods. J. Clean. Prod..

[6] European Commission (2023). Study on the Critical Raw Materials for the EU 2023-Final Report. https://op.europa.eu/en/publication-detail/-/publication/57318397-fdd4-11ed-a05c-01aa75ed71a1/language-en.

[7] Lima A.T., Kirkelund G.M., Ntuli F., Ottosen L.M. (2022). Screening dilute sources of rare earth elements for their circular recovery. J. Geochem. Explor..

[8] Watari T., Nansai K., Nakajima K. (2021). Major metals demand, supply, and environmental impacts to 2100: A critical review. Resour. Conserv. Recycl..

[9] Calvo G., Mudd G., Valero A., Valero A. (2016). Decreasing ore grades in global metallic mining: A theoretical issue or a global reality?. Resources.

[10] Magdalena R., Valero A., Valero A. (2021). Mining energy consumption as a function of ore grade decline: The case of lead and zinc. J. Sustain. Min..

[11] Izatt R.M., Izatt S.R., Bruening R.L., Izatt N.E., Moyer B.A. (2014). Challenges to achievement of metal sustainability in our high-tech society. Chem. Soc. Rev..

[12] Budhathoki R., Vaïsänen A. (2016). Particle size based recovery of phosphorus from combined peat and wood fly ash for forest fertilization. Fuel Process. Technol..

[13] Ayobami A.B. (2021). Performance of wood bottom ash in cement-based applications and comparison with other selected ashes: Overview. Resour. Conserv. Recycl..

[14] Kirkelund G.M., Damoe A.J., Ottosen L.M. (2013). Electrodialytic removal of Cd from biomass combustion fly ash suspensions. J. Hazard. Mater..

[15] Wolffers M., Weibel G., Eggenberger U. (2021). Waste wood fly ash treatment in Switzerland—Effects of co-processing with fly ash from municipal solid waste on Cr(VI) reduction and heavy metal recovery. Processes.

[16] Funari V., Bokhari S.N.H., Vigliotti L., Meisel T., Braga R. (2016). The rare earth elements in municipal solid waste incinerators ash and promising tools for their prospecting. J. Hazard. Mater..

[17] Vassilev S.V., Vassileva C.G. (2020). Contents and associations of rare earth elements and yttrium in biomass ashes. Fuel.

[18] Wang J., Guo M., Liu M., Wei X. (2020). Long-term outlook for global rare earth production. Resour. Policy.

[19] Alonso E., Sherman A.M., Wallington T.J., Everson M.P., Field F.R., Roth R., Kirchain R.E. (2012). Evaluating rare earth element availability a case with revolutionary demand from clean technologies supporting information. Environ. Sci. Technol..

[20] Binnemans K., Jones P.T., Blanpain B., Van Gerven T., Yang Y., Walton A., Buchert M. (2013). Recycling of rare earths: A critical review. J. Clean. Prod..

[21] Ebbers B., Ottosen L.M., Jensen P.E. (2015). Comparison of two different electrodialytic cells for separation of phosphorus and heavy metals from sewage sludge ash. Chemosphere.

[22] Liang S., Chen H., Zeng X., Li Z., Yu W., Xiao K., Hu J., Hou H., Liu B., Tao S. (2019). A comparison between sulfuric acid and oxalic acid leaching with subsequent purification and precipitation for phosphorus recovery from sewage sludge incineration ash. Water Res..

[23] Ottosen L.M., Kirkelund G.M., Jensen P.E. (2013). Extracting phosphorous from incinerated sewage sludge ash rich in iron or aluminum. Chemosphere.

[24] Wang Q., Li J.-S., Tang P., Fang L., Poon C.S. (2018). Sustainable reclamation of phosphorus from incinerated sewage sludge ash as value-added struvite by chemical extraction, purification and crystallization. J. Clean. Prod..

[25] Li J., Chen Z., Wang Q., Fang L., Xue Q., Cheeseman C.R., Donatello S., Liu L., Poon C.S. (2018). Change in re-use value of incinerated sewage sludge ash due to chemical extraction of phosphorus. Waste Manag..

[26] Hjelmar O., Hyks J., Korpisjärvi K., Wahlström M., Grönholm R. (2022). BAT (Best Available Techniques) for Combustion and Incineration Residues in a Circular Economy. https://pub.norden.org/temanord2022-542/.

[27] Tang J., Su M., Wu Q., Wei L., Wang N., Xiao E., Zhang H., Wei Y., Liu Y., Ekberg C. (2019). Highly efficient recovery and clean-up of four heavy metals from MSWI fly ash by integrating leaching, selective extraction and adsorption. J. Clean. Prod..

[28] Berra M., Mangialardi T., Paolini A.E. (2015). Reuse of woody biomass fly ash in cement-based materials. Constr. Build. Mater..

[29] Maresca A., Hyks J., Astrup T.F. (2017). Recirculation of biomass ashes onto forest soils: Ash composition, mineralogy and leaching properties. Waste Manag..

[30] Allegrini E., Maresca A., Emil M., Sommer M., Boldrin A., Fruergaard T. (2014). Quantification of the resource recovery potential of municipal solid waste incineration bottom ashes. Waste Manag..

[31] Gupta D.K., Chatterjee S., Datta S., Veer V., Walther C. (2014). Role of phosphate fertilizers in heavy metal uptake and detoxification of toxic metals. Chemosphere.

[32] Swiss Federal Council (2022). Ordinance on the Avoidance and the Disposal of Waste (Waste Ordinance, ADWO). https://www.fedlex.admin.ch/eli/cc/2015/891/en#chap_3/sec_3.

[33] (2022). Fertilizer Act 711/2022—Original Regulations. https://www.finlex.fi/fi/laki/alkup/2022/20220711.

[34] Zhai J., Burke I.T., Stewart D.I. (2022). Potential reuse options for biomass combustion ash as affected by the persistent organic pollutants (POPs) content. J. Hazard. Mater. Adv..

[35] Al-Ghouti M.A., Khan M., Nasser M.S., Al-Saad K., Heng O.E. (2021). A novel method for metals extraction from municipal solid waste using a microwave-assisted acid extraction. J. Clean. Prod..

[36] Zhu Y., Zhai Y., Li S., Liu X., Wang B., Liu X., Fan Y., Shi H., Li C., Zhu Y. (2022). Thermal treatment of sewage sludge: A comparative review of the conversion principle, recovery methods and bioavailability-predicting of phosphorus. Chemosphere.

[37] Abis M., Calmano W., Kuchta K. (2018). Innovative technologies for phosphorus recovery from sewage sludge ash. Detritus.

[38] Fang L., Li J.-S., Guo M.Z., Cheeseman C.R., Tsang D.C.W., Donatello S., Poon C.S. (2018). Phosphorus recovery and leaching of trace elements from incinerated sewage sludge ash (ISSA). Chemosphere.

[39] Li J., Tsang D.C.W., Wang Q., Fang L., Xue Q., Poon C.S. (2017). Fate of metals before and after chemical extraction of incinerated sewage sludge ash. Chemosphere.

[40] Weibel G., Eggenberger U., Kulik D.A., Hummel W., Schlumberger S., Klink W., Fisch M., Mäder U.K. (2018). Extraction of heavy metals from MSWI fly ash using hydrochloric acid and sodium chloride solution. Waste Manag..

[41] Xu H., He P., Gu W., Wang G., Shao L. (2012). Recovery of phosphorus as struvite from sewage sludge ash. J. Environ. Sci..

[42] Rahmani A., Thapa R., Aalto J.-M., Turhanen P., Vepsäläinen J., Lehto V.-P., Riikonen J. (2022). Functionalized nanoporous silicon for extraction of Sc from a leach solution. Hydrometallurgy.

[43] Fang L., Li J.-S., Donatello S., Cheeseman C.R., Wang Q., Poon C.S., Tsang D.C. (2018). Recovery of phosphorus from incinerated sewage sludge ash by combined two-step extraction and selective precipitation. Chem. Eng. J..

[44] Tang J., Steenari B.M. (2015). Solvent extraction separation of copper and zinc from MSWI fly ash leachates. Waste Manag..

[45] Turhanen P.A., Vepsäläinen J.J., Peräniemi S. (2015). Advanced material and approach for metal ions removal from aqueous solutions. Sci. Rep..

[46] Zalupski P.R., Chiarizia R., Jensen M.P., Herlinger A. (2006). Metal extraction by sulfur-containing symmetrically-substituted bisphosphonic acids. Part I. P,P′-di(2-ethylhexyl) methylenebisthio-phosphonic acid. Solvent Extr. Ion Exch..

[47] Lynn C.J., Dhir R.K., Ghataora G.S., West R.P. (2015). Sewage sludge ash characteristics and potential for use in concrete. Constr. Build. Mater..

[48] Krüger O., Grabner A., Adam C. (2014). Complete survey of German sewage sludge ash. Environ. Sci. Technol..

[49] Tang P., Xuan D., Li J., Cheng H.W., Poon C.S., Tsang D.C.W. (2020). Investigation of cold bonded lightweight aggregates produced with incineration sewage sludge ash (ISSA) and cementitious waste. J. Clean. Prod..

[50] Tian Y., Wang R., Luo Z., Wang R., Yang F., Wang Z., Shu J., Chen M. (2020). Heavy metals removing from municipal solid waste incineration fly ashes by electric field-enhanced washing. Materials.

[51] Yen C.-P., Zhou S.-Y., Shen Y.-H. (2020). The recovery of Ca and Zn from the municipal solid waste incinerator fly ash. Sustainability.

[52] Fang L., Wang Q., Li J.-S., Poon C.S., Cheeseman C.R., Donatello S., Tsang D.C.W. (2021). Feasibility of wet-extraction of phosphorus from incinerated sewage sludge ash (ISSA) for phosphate fertilizer production: A critical review. Crit. Rev. Environ. Sci. Technol..

[53] Meng X., Huang Q., Xu J., Gao H., Yan J. (2019). A review of phosphorus recovery from different thermal treatment products of sewage sludge. Waste Dispos. Sustain. Energy.

[54] Havukainen J., Nguyen M.T., Hermann L., Horttanainen M., Mikkilä M., Deviatkin I., Linnanen L. (2016). Potential of phosphorus recovery from sewage sludge and manure ash by thermochemical treatment. Waste Manag..

[55] Fraissler G., Joller M., Mattenberger H., Brunner T., Obernberger I. (2009). Thermodynamic equilibrium calculations concerning the removal of heavy metals from sewage sludge ash by chlorination. Chem. Eng. Process..

[56] Mattenberger H., Fraissler G., Brunner T., Herk P., Hermann L., Obernberger I. (2008). Sewage sludge ash to phosphorus fertiliser: Variables influencing heavy metal removal during thermochemical treatment. Waste Manag..

[57] Guedes P., Couto N., Ottosen L.M., Ribeiro A.B. (2014). Phosphorus recovery from sewage sludge ash through an electrodialytic process. Waste Manag..

[58] Ottosen L.M., Jensen P.E., Kirkelund G.M. (2016). Phosphorous recovery from sewage sludge ash suspended in water in a two-compartment electrodialytic cell. Waste Manag..

[59] Ottosen L.M., Bertelsen I.M.G., Jensen P.E., Kirkelund G.M. (2020). Sewage sludge ash as resource for phosphorous and material for clay brick manufacturing. Constr. Build. Mater..

[60] Villen-Guzman M., Guedes P., Couto N., Ottosen L.M., Ribeiro A.B., Rodriguez-Maroto J.M. (2018). Electrodialytic phosphorus recovery from sewage sludge ash under kinetic control. Electrochim. Acta.

[61] Righi C., Lancellotii I., Barbieri L., Kirkelund G.M. (2022). Benefits of pre-treating MSWI fly ash before alkali-activation. Sustain. Chem. Pharm..

[62] Oliveira V., Labrincha J., Dias-Ferreira C. (2018). Extraction of phosphorus and struvite production from the anaerobically digested organic fraction of municipal solid waste. J. Environ. Chem. Eng..

[63] (2022). Ash2Phos. A Circular Solution for Phosphorus Fertiliser. https://www.easymining.se/technologies/ash2phos.

[64] Ottosen L.M., Thornberg D., Cohen Y., Stiernström S. (2022). Utilization of acid-washed sewage sludge ash as sand or cement replacement in concrete. Resour. Conserv. Recycl..

[65] Quina M.J., Bontempi E., Bogush A., Schlumberger S., Weibel G., Braga R., Funari V., Hyks J., Rasmussen E., Lederer J. (2018). Technologies for the management of MSW incineration ashes from gas cleaning: New perspectives on recovery of secondary raw materials and circular economy. Sci. Total Environ..

[66] Schlumberger S., Schuster M., Ringmann S., Koralewska R. (2007). Recovery of high purity zinc from filter ash produced during the thermal treatment of waste and inerting of residual materials. Waste Manag. Res..

[67] HaloSep (2022). Pure Separation. https://www.halosep.com/.

[68] Karlfeldt Fedje K., Andersson S. (2020). Zinc recovery from Waste-to-Energy fly ash—A pilot test study. Waste Manag..

[69] (2022). Smart City Sweden. https://smartcitysweden.com/renova-is-building-for-the-worlds-unique-metal-recycling/.

[70] Ottosen L.M., Kirkelund G.M., Jensen P.E., Pedersen K.B. (2023). Extraction of Phosphorus from Sewage Sludge Ash-Influence of Process Variables on the Electrodialytic Process. Sustainability.

[71] Donatello S., Tong D., Cheeseman C. (2010). Production of technical grade phosphoric acid from incinerator sewage sludge ash (ISSA). Waste Manag..

[72] Xu Y., Zhang L., Chen J., Liu T., Li N., Xu J., Yin W., Li D., Zhang Y., Zhou X. (2023). Phosphorus recovery from sewage sludge ash (SSA): An integrated technical, environmental and economic assessment of wet-chemical and thermochemical methods. J. Environ. Manag..

[73] Eagle L., Rechberger H., Krampe J., Zessner M. (2016). Phosphorus recovery from municipal wastewater: An integrated comparative technological, environmental and economic assessment of P recovery technologies. Sci. Total Environ..

[74] Abramov S., He J., Wimmer D., Lemloh M.L., Muehe E.M., Gann B., Roehm E., Kirchhof R., Babechuk M.G., Schoenberg R. (2018). Heavy metal mobility and valuable contents of processed municipal solid waste incineration residues from Southwestern Germany. Waste Manag..

[75] Zhuge Y., Duan W., Liu Y. (2022). Utilization of wood waste ash in green concrete production. Sustainable Concrete Made with Ashes and Dust from Different Sources: Materials, Properties and Applications.

[76] De Rossi A., Simão L., Ribeiro M.J., Hotza D., Moreira R.F.P.M. (2020). Study of cure conditions effect on the properties of wood biomass fly ash geopolymers. J. Mater. Res. Technol..

[77] Luukkonen T. (2022). Alkali activation of water and wastewater sludges: Solidification/stabilization and potential aluminosilicate precursors. Development in Waste Water Treatment Research and Processes.

[78] Krejcirikova B., Ottosen L.M., Kirkelund G.M., Rode C., Peuhkuri R. (2019). Characterization of sewage sludge ash and its effect on moisture physics of mortar. J. Build. Eng..

[79] Carević I., Štirmer N., Trkmić M., Jurić K.K. (2020). Leaching characteristics of wood biomass fly ash cement composites. Appl. Sci..

[80] Carević I., Baričević A., Štirmer N., Šantek Bajto J. (2020). Correlation between physical and chemical properties of wood biomass ash and cement composites performances. Constr. Build. Mater..

[81] Chowdhury S., Mishra M., Suganya O. (2015). The incorporation of wood waste ash as a partial cement replacement material for making structural grade concrete: An overview. Ain Shams Eng. J..

[82] Gabrijel I., Rukavina M.J., Štirmer N. (2021). Influence of wood fly ash on concrete properties through filling effect mechanism. Materials.

[83] Milovanović B., Štirmer N., Carević I., Baričević A. (2019). Wood biomass ash as a raw material in concrete industry. Gradjevinar.

[84] Lothenbach B., Scrivener K., Hooton R.D. (2011). Supplementary cementitious materials. Cem. Concr. Res..

[85] Ashraf W. (2016). Carbonation of cement-based materials: Challenges and opportunities. Constr. Build. Mater..

[86] Koch R., Sailer G., Paczkowski S., Pelz S., Poetsch J., Müller J. (2021). Lab-scale carbonation of wood ash for CO_2_-sequestration. Energies.

[87] Li L., Wu M. (2022). An overview of utilizing CO_2_ for accelerated carbonation treatment in the concrete industry. J. CO2 Util..

[88] Tominc S., Ducman V. (2023). Methodology for Evaluating the CO_2_ Sequestration Capacity of Waste Ashes. Materials.

[89] Tripathi N., Hills C.D., Singh R.S., Singh J.S. (2020). Offsetting anthropogenic carbon emissions from biomass waste and mineralised carbon dioxide. Sci. Rep..

[90] Winnefeld F., Leemann A., German A., Lothenbach B. (2022). CO_2_ storage in cement and concrete by mineral carbonation. Curr. Opin. Green Sustain. Chem..

[91] (2019). Regulation (EU) 2019/1009 of the European Parliament and of the Council of 5 June 2019. Official Journal of the European Union. https://eur-lex.europa.eu/legal-content/EN/TXT/PDF/?uri=CELEX:32019R1009&from=EN.

[92] Kappel A., Viader R.P., Kowalski K.P., Kirkelund G.M., Ottosen L.M. (2018). Utilisation of electrodialytically treated sewage sludge ash in mortar. Waste Biomass Valorization.

[93] Donatello S., Freeman-Pask A., Tyrer M., Cheeseman C.R. (2010). Effect of milling and acid washing on the pozzolanic activity of incinerator sewage sludge ash. Cem. Concr. Compos..

[94] Istuque D.B., Soriano L., Akasaki J.L., Melges J.L.P., Borrachero M.V., Monzó J., Payá J., Tashima M. (2019). Effect of sewage sludge ash on mechanical and microstructural properties of geopolymers based on metakaolin. Constr. Build. Mater..

[95] Castro-Gomes J., Sedira N., Grünhäuser Soares E. (2022). Feasibility for Alkali-Activation of a Sewage Sludge Ash (SSA). https://www.researchgate.net/publication/359439591_Feasibility_for_alkali-activation_of_a_Sewage_Sludge_Ash_SSA.

[96] Chen W., Klupsch E., Kirkelund G.M., Jensen P.E., Ottosen L.M., Dias-Ferreira C. (2018). WASTES—Solutions, Treatments and Opportunities II: Selected Papers from the 4th Edition of the International Conference on Wastes: Solutions Treatments and Opportunities.

[97] Al-Ghouti M.A., Khan M., Nasser M.S., Al-Saad K., Heng O.E. (2021). Recent advances and applications of municipal solid wastes bottom and fly ashes: Insights into sustainable management and conservation of resources. Environ. Technol. Innov..

[98] Das S., Lee S.H., Kumar P., Kim K.H., Lee S.S., Bhattacharya S.S. (2019). Solid waste management: Scope and the challenge of sustainability. J. Clean. Prod..

[99] Fontseré Obis M., Germain P., Bouzahzah H., Richioud A., Benbelkacem H. (2017). The effect of the origin of MSWI bottom ash on the H2S elimination from landfill biogas. Waste Manag..

[100] Chen B., Perumal P., Illikainen M., Ye G. (2023). A review on the utilization of municipal solid waste incineration (MSWI) bottom ash as a mineral resource for construction materials. J. Build. Eng..

[101] Liu J., Hu L., Tang L., Ren J. (2021). Utilisation of municipal solid waste incinerator (MSWI) fly ash with metakaolin for preparation of alkali-activated cementitious material. J. Hazard. Mater..

[102] Pérez-Martínez S., Giro-Paloma J., Maldonado-Alameda A., Formosa J., Queralt I., Chimenos J.M. (2019). Characterisation and partition of valuable metals from WEEE in weathered municipal solid waste incineration bottom ash, with a view to recovering. J. Clean. Prod..

[103] Balaguera A., Carvajal G.I., Albertí J., Fullana-i-Palmer P. (2018). Life cycle assessment of road construction alternative materials: A literature review. Resour. Conserv. Recycl..

[104] Lam C.H.K., Ip A.W.M., Barford J.P., McKay G. (2010). Use of incineration MSW ash: A review. Sustainability.

[105] Li Y., Hao L., Chen X. (2016). Analysis of MSWI Bottom Ash Reused as Alternative Material for Cement Production. Procedia Environ. Sci..

[106] Parés Viader R., Jensen P.E., Ottosen L.M. (2017). Electrodialytic remediation of municipal solid waste incineration residues using different membranes. Chemosphere.

[107] Sekito T., Dote Y., Onoue K., Sakanakura H., Nakamura K. (2014). Characteristics of element distributions in an MSW ash melting treatment system. Waste Manag..

[108] Kaderides K., Papaoikonomou L., Serafim M., Goula A.M. (2019). Microwave-assisted extraction of phenolics from pomegranate peels: Optimization, kinetics, and comparison with ultrasounds extraction. Chem. Eng. Process. Process Intensif..

[109] Rahmati S., Abdullah A., Kang O.L. (2019). Effects of different microwave intensity on the extraction yield and physicochemical properties of pectin from dragon fruit (Hylocereus polyrhizus) peels. Bioact. Carbohydr. Diet. Fibre.

[110] Su D.-L., Li P.-J., Quek S.Y., Huang Z.-Q., Yuan Y.-J., Li G.-Y., Shan Y. (2019). Efficient extraction and characterization of pectin from orange peel by a combined surfactant and microwave assisted process. Food Chem..

[111] Zhan X., Kirkelund G.M. (2021). Electrodialytic remediation of municipal solid waste incineration fly ash as pre-treatment before geopolymerisation with coal fly ash. J. Hazard. Mater..

[112] Łach M., Mierzwiński D., Korniejenko K., Mikuła J., Hebda M. (2018). Geopolymers as a material suitable for immobilization of fly ash from municipal waste incineration plants. J. Air Waste Manag. Assoc..

[113] Pan S., Ding J., Peng Y., Lu S., Li X. (2022). Investigation of mechanochemically treated municipal solid waste incineration fly ash as replacement for cement. Energies.

[114] Ebert B.A.R., Kirkelund G.M. (2022). Effects of chlorides and sulphates on heavy metal leaching from mortar with raw and electrodialytically treated MSWI fly ash. Waste Biomass Valorization.

[115] Kirkelund G.M., Skevi L., Ottosen L.M. (2020). Electrodialytically treated MSWI fly ash use in clay bricks. Constr. Build. Mater..

[116] Sun J., Zhou H., Jiang H., Zhang W., Mao L. (2021). Recycling municipal solid waste incineration fly ash in fired bricks: An evaluation of physical-mechanical and environmental properties. Constr. Build. Mater..

[117] Voisniene V., Kizinievic O., Kizinievic V. (2019). Feasibility study of using clay bricks made from municipal solid waste incinerator (MSWI) fly ash. IOP Conf. Ser. Mater. Sci. Eng..

[118] Carević I., Serdar M., Štirmer N., Ukrainczyk N. (2019). Preliminary screening of wood biomass ashes for partial resources replacements in cementitious materials. J. Clean. Prod..

[119] Ukrainczyk N., Vrbos N., Koenders E.A.B. (2016). Reuse of woody biomass ash waste in cementitious materials. Chem. Biochem. Eng. Q..

[120] Royo J., Canalís P., Quintana D. (2022). Chemical study of bottom ash sintering in combustion of pelletized residual agricultural biomass. Fuel.

[121] Khan A.A., de Jong W., Jansens P.J., Spliethoff H. (2009). Biomass combustion in fluidized bed boilers: Potential problems and remedies. Fuel Process. Technol..

[122] Cheah C.B., Ramli M. (2011). The implementation of wood waste ash as a partial cement replacement material in the production of structural grade concrete and mortar: An overview. Resour. Conserv. Recycl..

[123] Mu L., Li T., Zuo S., Yin H., Dong M. (2022). Effect of leaching pretreatment on the inhibition of slagging/sintering of aquatic biomass: Ash transformation behavior based on experimental and equilibrium evaluation. Fuel.

[124] Guo Q., Yan B., Hu Y., Guo X., Wu W., Cheng Z., Chen G., Hou L. (2023). A novel reutilization of ash from biomass gasification process: Feasibility and products improvement analysis. Fuel.

[125] Drljaca D., Vukic L., Dragic D., Borkovic A., Botic T., Dugic P., Papuga S.V., Šolić M.D., Maletić S.P., Gvero P.M. (2022). Leaching of heavy metals from wood biomass ash, before and after binding in cement composite. J. Serbian Chem. Soc..

[126] Akinyemi B.A., Dai C. (2020). Development of banana fibers and wood bottom ash modified cement mortars. Constr. Build. Mater..

[127] Elinwa A.U. (2006). Effect of addition of sawdust ash to clay bricks. Civ. Eng. Environ. Syst..

[128] Hafez R.D.A., Tayeh B.A., Abd-Al Ftah R.O. (2022). Development and evaluation of green fired clay bricks using industrial and agricultural wastes. Case Stud. Constr. Mater..

[129] Gunning P., Hills C., Antemir A., Carey P. Novel approaches to the valorisation of ashes using aggregation by carbonation. Proceedings of the 2nd International Slag Valorisation Symposium 18-20 April 2011.

[130] Vassilev S.V., Vassileva C.G., Petrova N.L. (2021). Mineral carbonation of biomass ashes in relation to their CO_2_ capture and storage potential. ACS Omega.

[131] Ke X., Baki V.A., Skevi L. (2023). Mechanochemical activation for improving the direct mineral carbonation efficiency and capacity of a timber biomass ash. J. CO2 Util..

[132] Abdulkareem O.A., Ramli M., Matthews J.C. (2019). Production of geopolymer mortar system containing high calcium biomass wood ash as a partial substitution to fly ash: An early age evaluation. Compos. Part B Eng..

[133] Zhang Z., Wang H., Provis J.L. (2012). Quantitative study of the reactivity of fly ash in geopolymerization by FTIR. J. Sustain. Cem. Based Mater..

[134] da Costa T.P., Quinteiro P., Tarelho L.A.C., Arroja L., Dias A.C. (2020). Life cycle assessment of woody biomass ash for soil amelioration. Waste Manag..

[135] Pasquali M., Zanoletti A., Benassi L., Federici S., Depero L.E., Bontempi E. (2018). Stabilized biomass ash as a sustainable substitute for commercial P-fertilizers. Land Degrad. Dev..

